# Developing a Music-Based Mobile Health Intervention for Parents and Staff in Neonatal Care and Beyond: Online Focus Group Study

**DOI:** 10.2196/85344

**Published:** 2026-07-29

**Authors:** Julia Seinsche, Bettina Schwind, Jörg Haslbeck, Rosa MS Visscher, Dirk Bassler, Friederike B Haslbeck

**Affiliations:** 1 Careum School of Health Kalaidos University of Applied Sciences Zurich Switzerland; 2 Institute of Biomedical Ethics and History of Medicine University of Zurich Zurich Switzerland; 3 Chair of Public Health and Health Services Research Institute for Medical Information Processing, Biometry, and Epidemiology Ludwig-Maximilians-Universität München Munich Germany; 4 Department of Neonatology University Hospital of Zurich Zurich Switzerland

**Keywords:** creative music therapy, family-integrated care, focus group, mind mapping, mobile health, neonatology, preterm infants, usability, user needs

## Abstract

**Background:**

Creative music therapy (CMT) in preterm infants has been associated with improved health outcomes in both parents and infants while promoting parent-infant bonding. However, access to CMT is limited.

**Objective:**

This study aimed to explore the needs and preferences of parents of preterm infants and health care professionals as a first step in developing a CMT-based mobile health (mHealth) intervention.

**Methods:**

An iterative qualitative study combining multistakeholder online focus groups with mind mapping. Map contributions were analyzed using reflexive thematic analysis.

**Results:**

A total of 20 participants (7 parents, 1 grandparent, and 12 health care professionals) contributed to creating the mind map, offering insights into the identified five themes: (1) aims of the mHealth intervention, (2) desired functions and design, (3) content, (4) important factors for acceptability, and (5) usability beyond the neonatal intensive care unit.

**Conclusions:**

The study highlights critical considerations for designing a CMT-based mHealth intervention. The in-depth user insights gathered will inform iterative development and refinement of the intervention, which will be evaluated in future usability, feasibility, and effectiveness studies.

## Introduction

Globally, an estimated 13.4 million babies are born preterm, which is 9.9% of all births, and this number has been stable over the past decade, with only a minimal decline [1]. Preterm births, defined as delivery before 37 weeks of gestation, are a major cause of newborn mortality and are associated with long-term neurodevelopmental impairments [2-4]. Neurodevelopmental impairments may be triggered through stress responses due to unexpected parent-infant separation and trauma [5], auditory deprivation (eg, lack of exposure to intrauterine maternal heartbeat and the mother’s voice), and the overstimulating, stressful, noisy neonatal intensive care unit (NICU) context [6,7].

The traumatic nature of preterm birth, combined with uncertainties regarding the infant’s health and future and the stressful situation in the NICU, profoundly affects parents as well, resulting in increased rates of anxiety [3,8], depression [3,8], posttraumatic stress [8], and feelings of helplessness [9,10] or disempowerment [11]. Such psychological issues and the physical separation of parents and infants can impact the parent-infant relationship, interaction, and the formation of a secure attachment—an emotional bond, which is formed in the first 18 months of life [3,12] and is associated with the infant’s later socioemotional, behavioral, and cognitive development [3,5,13].

Conversely, in NICUs supporting active parental involvement, such as applying kangaroo-care, improved outcomes have been observed, including reductions in infant mortality and morbidity [14]. This underscores the importance of family-centered care, including free parental access to the NICU and psychological support for parents [15], as well as family-integrating care models with parents being acknowledged as primary caregivers [16].

Building on this foundation, further approaches promoting active parental involvement in the NICU include interventions such as family-integrating creative music therapy (CMT), which emphasizes meaningful multimodal stimulation. CMT uses infant-directed, lullaby-style singing to soothe and gently stimulate infants, while empowering parents through active involvement in the therapeutic process [17-19], that is, music therapy during skin-to-skin contact to foster bidirectional relaxation, attachment, and bonding. Throughout the music therapy process, parents are guided and empowered to recognize their infants’ cues and tailor their responses to the infants’ state and needs, while humming, speaking, and/or singing in a responsive way. CMT is associated with reduced parental stress and anxiety [20,21] and improved neurodevelopmental outcomes [5,18], as well as parent-infant bonding [20]. Moreover, previous systematic reviews demonstrated various beneficial effects of music on physiological parameters such as heart and respiratory rate, oral feeding, behavior, and stress in preterm infants [21,22], and a Cochrane review [23] confirmed an overall substantial beneficial effect of music and vocal interventions on the preterm infants‘ heart rate during and after the intervention. Despite these promising outcomes, access to CMT in NICUs is limited [24] due to the limited availability of therapists [25-27].

One way to address this limited access to CMT in the NICU is the use of mobile health (mHealth) interventions, delivered via an app or SMS text messages. They are increasingly applied in health care delivery and offer major advantages such as cost-effectiveness and accessibility compared to traditional face-to-face interventions and usual care [28-31]. Numerous mHealth interventions for parents exist [32], targeting pregnancy and postpartum care [33-36], parenting [35,37,38], and risk of preterm birth [39,40]. However, there is a notable lack of comprehensive mHealth interventions specifically designed for parents of preterm infants and infants born with serious illness, and, to the best of our knowledge, no music therapy-based mHealth intervention exists.

Another important consideration is that commercially available apps may not have been developed and tested with the same rigor as those created in research settings [41]. This raises concerns about their reliability, feasibility, usability, and real-world impact.

Therefore, a research and development (R&D) project was initiated [42]. It aims to develop a CMT-based mHealth intervention delivered through an app to support, coach, and motivate parents of preterm infants and health care professionals in the NICU and later at home to use their voice and music. This, in turn, will improve well-being and the parent-infant bond. The R&D project is based on a user-centered design (UCD) approach—an iterative process that involves end users throughout the whole development process to continuously refine and reshape the intervention’s design [43,44]. The UCD approach is complemented by a broader human-centered perspective, which considers emotional, social, and contextual realities in which the intervention will be used. This is especially relevant in sensitive settings such as the NICU [45,46].

This study represents the first working package of this project [42], aiming to explore primary (parents) and secondary (health care professionals) end users’ needs and preferences toward such an mHealth intervention. Its overall objective was to provide a basis for further iterative co-design steps, such as developing a prototype of the mHealth intervention, rapid usability testing, effectiveness evaluation, and implementation.

## Methods

### Study Design and Setting

The study reported in this article is an iterative qualitative study, combining multistakeholder online focus groups with mind mapping and prioritization of results to reach saturation. Study reporting was conducted in compliance with the COREQ (Consolidated Criteria for Reporting Qualitative Research) [47].

The whole R&D project is a collaboration between the University Hospital Zurich Department of Neonatology and the University of Zurich, with the Careum School of Health, part of the Kalaidos University of Applied Sciences, and amiamusica (a charity association dedicated to empowering preterm and high-risk newborns and their families through music). The project’s implementation partner is Pathmate Technologies, a company that develops coaching technologies combining medicine and behavioral psychology with data and artificial intelligence. The core research team comprises an interdisciplinary group of senior researchers who have conducted research in the fields of CMT in neonatal care, public health, care and caring, movement sciences, and health sciences and technology, and are experienced in participatory and transdisciplinary research.

### Ethical Considerations

The ethics committee of the Canton of Zurich reviewed the study and decided that it does not fall within the scope of the Human Research Act, which regulates research on human subjects in Switzerland (BASEC-Nr Req-2024-00635). The study was conducted in compliance with all national legal and regulatory requirements.

### Participants and Recruitment

Study participants were purposefully sampled, including parents and grandparents of preterm infants and health care professionals. We aimed to recruit German- and English-speaking mothers and fathers and, if possible, grandparents, with and without music therapy experience, all of whom experienced preterm birth at least 1 year prior to participation. We sought to include a variety of ages, genders, educational, socioeconomic, and migration backgrounds. Health care professionals, such as nurses, doctors, psychologists, and music therapists, were eligible if they had a minimum of 3 years of working experience in the NICU.

Recruitment was conducted through the parent association amiamusica, University Hospital Zurich, University of Zurich, as well as through professional neonatology and parent networks and foundations in Switzerland, Germany, and the United Kingdom. Potential participants were approached via email by BS, RMSV, FBH, and JS, and before participating in the study, they had to provide digital informed consent using the online survey tool Unipark (Tivian GmbH, 2024).

### Study Procedures

#### ECOUTER Approach

Study procedures followed the first step of a UCD process—user research and analysis—inspired by the ECOUTER (Employing Conceptual Schema for Policy and Translation Engagement in Research) approach proposed by Murtagh et al [48], which is based on iterative creation and coediting of mind maps by a diverse group of participants. The ECOUTER approach relies on flexibility and adaptability and aims to open up discourses. Its crucial advantage is that it can be conducted virtually, making it accessible to a broad range of people by overcoming temporal and physical limitations [48]. Based on the ECOUTER procedures and following previous research that implemented and described the approach [49], our study comprised three steps: (1) participant engagement, knowledge exchange, and prioritization of results; (2) thematic analysis of mind map contributions; and (3) result interpretation and development of recommendations.

#### Participant Engagement, Knowledge Exchange, and Prioritization of Results

**Figure 1:**
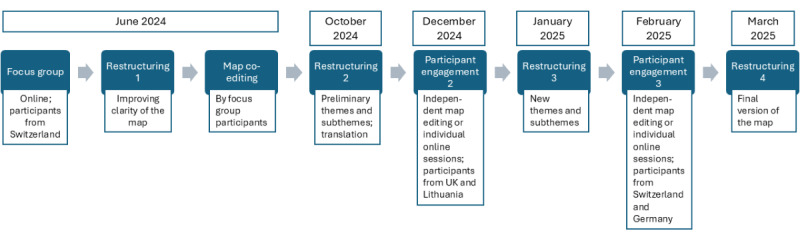
Study procedures: participant engagement and thematic map analysis.

A total of 3 groups of participants took part in this study (Figure 1). First, a 2-hour online focus group session was conducted via Zoom in June 2024 with participants from Switzerland. The session aimed to explore users’ needs following a focus group guide developed by BS and FBH. FBH moderated the focus groups, while 3 additional interdisciplinary study investigators (BS, RMSV, and JH) observed the online interactions, took reflective notes, and documented the discussions on the online whiteboard Miro using mind mapping. Afterward, participants were invited to comment on the map and mark the aspects they found most central with stickers.

This mind map served as a basis for two further rounds of participant engagement in December 2024 and February 2025, involving participants from the United Kingdom and Lithuania, and from Switzerland and Germany, respectively. All participants edited the mind map by adding their ideas and marking priorities—either independently on the online Whiteboard Miro or during facilitated individual online sessions with FBH and BS, JH, or JS. Participants were allowed to access the mind map as often as they preferred for a period of 1 week. All focus groups and online sessions were video recorded. We defined thematic saturation as the point at which interaction with the mind map ceased.

#### Thematic Analysis of Mind Map Contributions and Result Interpretation

The mind map was analyzed, inspired by the principles of reflexive thematic analysis as outlined by Braun and Clarke [50,51].

First, the initial version of the mind map, which was developed collaboratively during the first focus group session, was inspected and reviewed by each member of the research team to gain a deep understanding of the range of ideas and expressions contributed by parents and health care professionals. It then underwent four rounds of restructuring (Figure 1).

A major restructuring occurred in June 2024, immediately after the focus group session, focusing on improving the clarity and coherence of the map for subsequent commenting and prioritization by focus group participants. Another major revision was undertaken in October 2024 prior to translating and sharing the map with participants from the United Kingdom and Lithuania, with a focus on grouping related ideas and further developing and labeling preliminary themes and subthemes. Additional restructuring was conducted in January and March 2025 before and after the final round of participant engagement. The final version of the map can be found in Multimedia Appendix 1.

In each restructuring phase, themes and subthemes were initially developed by JS and BS and subsequently iteratively edited based on internal feedback loops, including both individual reflections and project team discussions. Themes and their labels were critically examined for internal coherence and distinctiveness. The team repeatedly cross-checked whether the emerging thematic structure reflected the diversity of participant input, while enhancing clarity. Unclear or overlapping themes were either refined or combined. Changes were made collaboratively until team consensus was reached, and no additional codes or themes emerged.

In addition to these 4 major rounds of restructuring, constant modifications by the research team occurred in parallel to participants adding annotations or stickers to enhance clarity and comprehensibility for others. Overall, this iterative coanalysis approach aimed to ensure transparency, thematic depth, and validity in theme development while maintaining the participatory nature of the data, which stemmed from the ECOUTER approach, with participants being allowed to add, relocate, and comment on mind map contributions.

The final step comprised the summary of the map structured by themes and subthemes, and their interpretation.

## Results

### Participants

The study involved 20 participants who actively contributed to creating the mind map (n=3 from the United Kingdom, n=2 from Lithuania, n=13 from Switzerland, and n=2 from Germany). A total of 7 of those participants were parents of at least 1 preterm infant, and 1 participant was a grandparent, while the group of health care professionals comprised medical doctors (n=3), music therapists (n=5), a psychologist (n=1), and nursing experts (n=2). One participant was both a psychologist and a music therapist. Two of the music therapists were also parents of a preterm infant, but were counted as professional experts as they were enrolled as such. All participants have completed tertiary education.

Table 1 presents the characteristics of the first 2 participant groups. To validate the findings from those groups and following the ECOUTER approach, which seeks to “open up” engagement iteratively and organically [48], the third and final round of participant involvement was conducted in a more open manner. Thus, no assignment of individual map contributions to specific participants was documented, and, as a result, detailed participant characteristics were only reliably collected for the first 2 groups.

**Table 1:** Characteristics of participant group 1 and group 2.

				Total		Group 1 (focus group)		Group 2 (online interviews/Miro board)
Family members								
	Participants, n			4		3		1
	Place of residence, n							
		Switzerland	3		3		N/A^a^	
		United Kingdom	1		N/A		1	
	Age (years), range			33-54		33-38		54
	Sex, n							
		Female	3		2		1	
		Male	1		1		0	
Experts								
	Participants, n			8		4		4
	Place of residence, n							
		Switzerland	4		4		N/A	
		United Kingdom	2		N/A		2	
		Lithuania	2		N/A		2	
	Age (years), range			39-53		43-53		39-45
	Sex, n							
		Female	7		3		4	
		Male	1		1		0	
	Profession, n							
		Medical doctor	2		1		1	
		Music therapist	4		1		3	
		Psychologist	1		1		0	
		Nursing expert	1		1		0	
	Work experience (years), range			2-25		15-25		2-15
	Work experience in the NICU^a^ (years), range			0.5-13		7-13		0.5-9

^a^N/A: not applicable.

^b^NICU: neonatal intensive care unit.

### Reflexive Thematic Analysis

**Figure 2:**
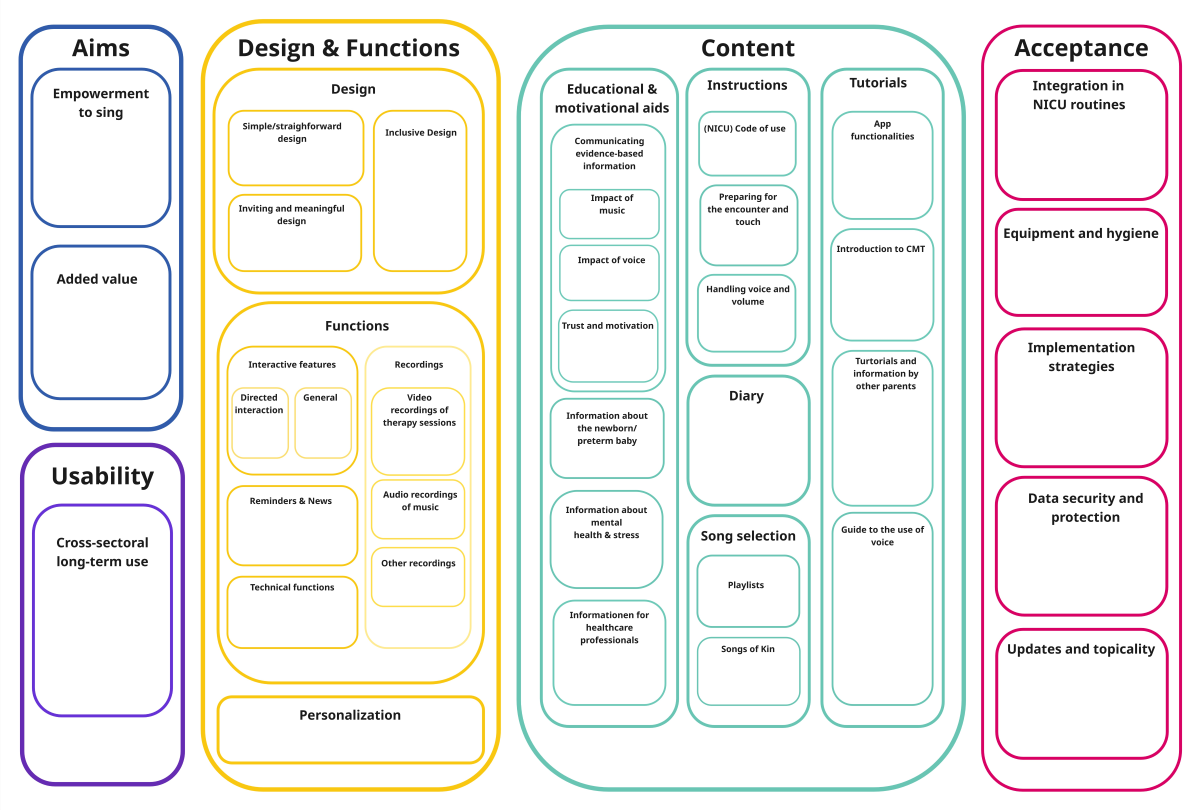
Overview of themes and subthemes after the final restructuring (restructuring 4). NICU: neonatal intensive care unit.

The reflexive thematic analysis of the board resulted in five themes describing (1) aims of the mHealth intervention, (2) desired functions and design, (3) content, (4) important factors for acceptability, and (5) how the intervention can be used beyond the NICU in ambulatory care. In the following, each theme and its subthemes are described with a focus, where applicable, on prioritized aspects. A graphical overview is provided in Figure 2, while a more detailed mind-map representation, including an indication of which themes were prioritized, can be found in Multimedia Appendix 1.

#### Theme 1: Aims of the Intervention

##### Overview

In this first theme, key goals and expectations toward the CMT-based mHealth intervention are described. A central priority of participants was to empower parents to sing to their infants and to benefit from the emotional, relational, and developmental advantages of CMT.

##### Subtheme: Empowerment to Sing

Participants emphasized the general importance of such an mHealth intervention for parents of preterm infants in the NICU and after discharge. Especially its relevance in NICU settings was highlighted, as they constitute an exposed restrictive space, likely inhibiting parents from singing. Therefore, the intervention’s primary goal should be to create a safe space, motivate and encourage parents to use their voices, as encapsulated in the slogan “Your voice is the most beautiful,” and ultimately enhance self-efficacy and empowerment. Among all participant groups, especially parents with lived NICU experience emphasized the intervention’s role in fostering empowerment in such a restrictive environment. Additionally, the intervention should promote physical and eye contact between parents and their children.

To overcome any inhibitions, participants underlined the importance of an easy introduction to singing without any pressure (“Just be a parent, not a singer”). Strategies include allowing parents to listen first and involving nurses to help create a more supportive and natural singing environment in the NICU.

##### Subtheme: Added Value

Participants emphasized the potential benefits of the planned CMT-based mHealth intervention, including facilitation of parent-infant bonding, promotion of a sense of security for preterm infants and infants born with serious illness since they are familiar with their parents’ voices from pregnancy (“Child feels safe, secured and connected with parents”), and improvement of music therapy adherence. It possibly could help parents to get in touch with peers and therapists and encourage self-care and self-efficacy through a low-threshold entry to CMT. This can help parents to relax, which, in turn, would positively affect their infants.

#### Theme 2: Design and Functions

##### Overview

This theme outlines design preferences, functional requirements, and personalization needs for the app through which the mHealth intervention is delivered.

##### Subtheme: Design

The subtheme design was particularly prioritized by participants. Key thematic design aspects included the need for a “simple, straightforward,” and aesthetically “inviting and meaningful design” that is “inclusive,” flexible, and respectful of the diversity of potential users.

Participants stressed that the app should be “simple and straightforward,” thus user-friendly, easy to navigate, and logically organized (“Keep it simple and sexy”). Potential users suggested the need for a clear and consistent structural design, for example, a tree-like organization allowing for a continuing progression through the various functions, and emphasized the importance of a simple and intuitive menu with direct selection of the main functions from anywhere. Overall, preventing future users from feeling overwhelmed by complexity was considered key.

Participants also emphasized an “inviting and meaningful design,” stressing that the target group must always be kept in mind, that is, parents “should feel addressed in their adult role,” which is why the app should not be too colorful. Nevertheless, the style of the app should not be clinical and sterile either, but was proposed to be visually appealing, using a color scheme that would evoke warmth and hospitality. The use of symbols such as the octopus—a global symbol for preterm births—was proposed.

“Inclusive design” refers to the need for barrier-free access, which was emphasized by participants. The app should be accessible to individuals with vision impairments, neurodivergence, learning disabilities, or limited technical skills. Suggested solutions include using neurodivergent-friendly fonts, simple language, and voice control. Additionally, the app should be multilingual, gender-neutral, and avoid biased languages and references to reach a wider audience, achieve international visibility, and overcome language barriers (“address different cultures yet keep it simple”).

##### Subtheme: Functions

In terms of functions, thematic aspects included “interactive features,” “recordings,” “reminders and news,” and “technical functions.”

Participants highlighted the importance of “interactive features,” which, for instance, enable direct, bidirectional communication between NICU professionals and parents through images, videos, messages, and phone calls, allowing parents to stay informed about their child’s development and receive updates while not being present (“being able to share beautiful moments with the children with parents as an achievement from care work [appreciation]”). It was also highlighted that parents could send questions to professionals for follow-up discussions on the ward. However, health care professionals recommended exercising caution to avoid raising unrealistic expectations among parents that health care providers could react upon queries at any time and raised the concern that not being able to account for parents’ worries might lead to insecurity and worry, thus leaving them alone with their fears. A parent forum for peer-to-peer communication was also suggested, allowing the exchange of pictures, videos, experiences, and mutual understanding during the time in the NICU and for long-term use at home. Carefully moderating such a forum would be crucial to prevent the spread of harmful information.

Additionally, participants recommended incorporating feedback options for the mHealth intervention to develop and improve constantly.

Another important topic was “video and audio recordings” of CMT sessions and parent-child interactions. These recordings should be both asynchronous for sharing with family and friends and synchronous for involving other family members in real time. Participants also suggested “recordings of lullabies that can be played so babies can hear their mother’s voice” and recordings of the baby’s heartbeat, especially for palliative care as a special memory of the infant.

The thematic aspect “reminders and news” referred to sending weekly evidence-based updates on the benefits of CMT, which was considered an option for reminding and motivating parents to engage with the app and use their voices continuously (“Motivation for the longer run: Sharing evidence [...] why it is important [health of the baby] along with reminders”).

Participants mentioned several “technical aspects” which should be considered, namely offline functionality, compatibility with different systems, the option to download all content “to easily transfer the great memories to the computer or save them on the mobile phone,” and support for multiple profiles under a single account (for families with more than one child).

##### Subtheme: Personalization

Participants voiced personalization of the app’s design, functions and intervention content to ensure accessibility and motivation. They suggested a gradual progression through various levels of experience with music, particularly for users who consider themselves unmusical (“Where do you meet someone who describes him/herself as untalented/unmusical [...] maybe via a questionnaire that records where he/she sees him/herself?”). Furthermore, the vocal range (eg, soprano or tenor) should be determined via an app function as a basis for personalized song recommendations.

#### Theme 3: Content on Music, Health, and Motivational Support

##### Overview

Participants articulated a range of content-related needs and expectations, emphasizing the app’s role as an educational and motivational support tool. The theme “content” comprises the subthemes “educational and motivational aids,” “instructions,” and “tutorials,” referring to practical and voice-use guidance, a “diary” to support memory-making, and assistance with “song selection” to build parents’ confidence.

##### Subtheme: Educational and Motivational Aids

This subtheme describes the communication of “evidence-based information,” “information about the newborn/preterm baby,” “information about mental health and stress,” and “information for health care professionals.”

It was deemed essential that the mHealth intervention include empowering messages and “evidence-based information” on the impact of voice, singing, and CMT on child development and parental mental health to strengthen health literacy (“Develop perspectives for parents. It won’t be like this forever”). “Information on preterm babies,” such as their hearing abilities, way of communication, and milestones of development, was considered valuable. However, it was emphasized to carefully avoid putting pressure on parents due to the varying developmental pace of preterm babies. Instead, parents could be encouraged to track their own personal milestones in the app.

Overall, trustworthy and reliable information was regarded as essential for fostering trust, motivation, and reducing stress resulting from the otherwise stressful search for reliable information. Additionally, motivation to use one’s own voice and singing could be enhanced by highlighting the intervention’s cost-effectiveness, safety, simplicity, and its significant impact on infants’ health, thereby encouraging parents to use their voices as a tool for self-care during moments of helplessness as highlighted in this quote: “Let parents know why music therapy is helpful for their children (inexpensive use of voice with a major impact on health of the baby); simple but very effective, does not do any harm and helps babies grow”.

According to study participants, “information for health care professionals” should comprise the same background knowledge on the benefits and impact of CMT as parents, as they may equally be unaware of its impact on newborns and parents, and additional specialist information supporting their interaction with parents (“NICU staff needs to know how families feel on NICU and how music therapy helps them”).

##### Subtheme: Instructions

The subtheme “instructions” was found important but not prioritized compared to the other themes. It comprises thematic aspects related to “NICU code of use,” “handling voice and volume,” and “preparing for the encounter and touch.”

Instructions on how to use the intervention (“the code of conduct”) in the NICU included guidelines on phone usage (frequency, duration, volume, and privacy issues) and a reminder that the app is not a substitute for parental presence. Participants recommended instructions on “handling voice and volume” to avoid noise and overstimulation, instead of therapeutic auditory stimulation. Learning how to read and observe the babies’ reactions and real-time resonance was considered key to this. Participants emphasized the importance of differentiating between newborns and premature babies in terms of their responses, as expressed in this note: “Newborns appear much more responsive. At the same time, parents need to be aware of the signs of excessive demands on premature babies.” A calm atmosphere and touch should be emphasized in the context of “preparing for the encounter and touch,” including ideas and possibilities on how to create a calm and private atmosphere in a noisy NICU.

##### Subtheme: Tutorials

A video demonstration of the app’s functionalities and introductory videos on voice and singing techniques were considered essential. Such videos could provide step-by-step guides for future users with limited technological abilities or cognitive disabilities. They were also considered as alternative learning means compared to text-based content only to meet the needs of different potential users and learners, particularly when it comes to singing and music. Participants favored including real-life videos to create a feeling of relatability for parents. Personal videos from other parents sharing their experiences with voice, music, and therapy were seen as additional valuable resources (“Knowing that others are doing it and how they are doing is [in terms of motivation/peer] lets me do this, too”). Such real-life videos may also serve as a means for reducing fear of error when singing and thus support effective learning.

Participants also considered it important to provide a slow, low-threshold introduction to using voice and CMT, starting with speaking, then humming, and finally singing to promote parents’ self-efficacy and overcome insecurities. Such gradual progression may support future users in progressing at their own pace, according to their skills and capabilities. Involving music therapists in this might make it even easier (“Creating a caring environment with a trusted person so as not to feel alone/overwhelmed when singing”). However, participants emphasized that gender differences should be considered, with men potentially requiring different types of encouragement compared to women. Overall, a gender inclusive design of tutorials was emphasized.

##### Subtheme: Diary

A digital memory box or diary function was considered a key added value, allowing parents to store and retrieve photos, videos, and musical moments even after NICU discharge. This feature was anticipated to motivate parents by showing highlights and milestones (“could have milestone markers attached to a photo or short video”), while also preserving memories, particularly for parents dealing with palliative situations (“Dealing with palliative moments from terminal phases as a video moment—a ‘treasure’ for parents”). Pictures of the baby were expected to encourage milk production and support breastfeeding. The diary function was considered particularly valuable, even among HCPs, as parents are already given diaries to document and remember their time in the NICU and their child’s development. Therefore, the diary function could be useful in promoting the app’s acceptance and adoption in clinical processes.

##### Subtheme: Song Selection

This subtheme comprised thematic aspects related to “playlists” and “song of kin.” In accordance with participants’ preference for personalization, they suggested offering “playlists” with songs tailored to different experience levels, including songs for beginners, to “regain confidence in your own voice (melody with soundtrack, karaoke, or sing-along). It was found important to provide song recommendations for different situations, such as calming or activating songs, and to include songs that could be sung along to or performed as karaoke, supporting the transition from humming to singing.

The implementation of “songs of kin” was found necessary, that is, songs identified as especially meaningful to the parents, which can “bring familiar cultural heritage to the forefront of personalized treatment” [52]. According to participants, the mHealth intervention should “give impulses to what could be a song of kin” and encourage parents to be creative in choosing their own songs, even if these, at first thought, might not be considered baby-friendly. This could empower families to “select songs in an environment, where there is little choice” in order to support feelings of control.

#### Theme 4: Acceptance

##### Overview

In contributions clustered under “acceptance,” participants discussed a range of practical and systemic factors that are crucial for the intervention’s successful implementation and long-term acceptance in the NICU context. These included “integration into existing clinical routines,” “implementation strategies,” and “equipment availability and hygiene.” Furthermore, participants highlighted the importance of “data security and protection” and “updates and topicality.” These aspects were mainly emphasized and strongly prioritized by health care professionals.

##### Subtheme: Integration in NICU Routines

For the intervention to be accepted, health care professionals considered it crucial to take both parents’ and their own needs into account and to integrate the intervention into existing NICU routines (“applicability in everyday life required”). For example, the diary function could align with the practice of creating a record of the newborn’s time in the NICU (“Keep it simple and combine it with existing information (...) from daily businesses”). One concern shared among participants was the time constraint of health care professionals, which must be considered during implementation. Therefore, the app should include features that simplify or substitute existing processes, rather than adding to the workload. Finally, given the clinical restriction of the use of mobile devices in the NICU, the app should have a clear, strict code of use.

##### Subtheme: Implementation Strategies

Appropriate promotional or implementation strategies were found to be important to boost acceptance of the intervention. These could include preliminary written information (eg, flyers) and personal information provided by music therapists (“It is important for parents to feel welcomed: there is someone [the therapist] who accompanies me. Sensitivity is required when making first contact”).

##### Subtheme: Equipment and Hygiene

In terms of acceptability, health care professionals stated that if tablets are provided by the hospitals, dedicated tablets for each bed would be needed to ensure accessibility and sufficient hygiene. Proper covers and disinfection should be in place to maintain cleanliness and prevent cross-contamination.

##### Subtheme: Data security and Protection

Data security and privacy are paramount due to the transfer and storage of sensitive clinical data and pictures of families and health care professionals (“Think about data protection for staff as well. If parents film and record, the personal rights of staff can also be violated. Involve the staff committee at an early stage”). In this regard, licensing was found to be highly relevant to increasing trustworthiness. Participants expressed a preference for the mHealth app to be free of charge, without internal advertising or subscription fees.

##### Subtheme: Updates and Topicality

Another subtheme was the need for continuous updates of the app. Participants highlighted that evolving needs, particularly those caused by generational shifts, necessitate regular updates (“continue to ensure the involvement of parents because of changing needs between different generations”). A feedback function for continuous improvement was therefore considered key. For example, today’s young parents may require gender-inclusive language. Additionally, adaptation possibilities should be considered for international intervention implementation.

#### Theme 5: Usability Beyond the NICU: Cross-Sectoral Long-Term Use

The final theme focused on the mHealth intervention’s application after discharge from the NICU, considering its cross-sectoral long-term use and sustainability. Key thematic aspects included “considering different developmental stages,” “using and integrating music at home,” “support services and networking possibilities for aftercare issues,” and the need to “make data sustainably accessible.” None of these aspects was prioritized by participants, but considered add-ons, which would be nice to have.

Overall, to ensure long-term usability, the intervention should be adaptable to “different developmental stages” and age, offering tailored solutions or support for each stage (“Visualize different phases of life and find answers”).

Participants suggested providing input and guidance on how to “use and integrate music” into everyday home routines over a more extended period, including both very simple and in-depth information. As during the NICU stay, the app should highlight everyday situations in which music can be used for a specific purpose, such as aiding sleep or animating children (“point out everyday situations the children can be calmed [eg, after a fall] or animated/motivated [eg, when brushing teeth] by music”), also when they are growing up (playful music games).

There was a strong emphasis on the need for ongoing support in the long-term care of preterm infants and on addressing later “aftercare issues” such as difficulties in school. Possibilities included peer-to-peer networking and access to external support services, such as specialized psychologists (“support services for later concerns, as after discharge, you are on your own”).

Finally, for long-term sustainability, participants proposed export options for all data, allowing parents to access their data and photos outside of the application and ensuring that the information remains “sustainably accessible” even without continued application use.

## Discussion

### Principal Findings

The thematic analysis of participant-generated inputs reveals a broad and multifaceted spectrum of user expectations across parents and professional groups for a CMT-based mHealth intervention as a means toward family-integrated care, which supports, coaches, and motivates parents of preterm infants in neonatal care and at home. Our findings underscore the need to develop digital solutions that focus on promoting parent-infant bonding and empowering parents to both sing to their infants and benefit from the emotional, relational, and developmental advantages of music therapy. Thereby, rather than focusing solely on technical functionalities, participants emphasized a diverse range of needs encompassing a straightforward and inclusive design, educational and motivational content, personalization of functions and content, social support and peer exchange, and features for cross-sectoral long-term use of the mHealth intervention. Health care professionals further stressed the importance of integrating the mHealth intervention in NICU routines to increase its acceptance. These needs reflect a broader understanding of mHealth tools as holistic systems that must consider not only instrumental use but also affective needs to engage users effectively.

The multidimensionality of user expectations beyond technical functionalities has long been recognized in diverse research, for instance on general user needs [53] in the technology acceptance model [54], the unified theory of technology acceptance [55], and in the consumption value theory, which posits that consumer choice is influenced by 5 types of value: functional, conditional, social, emotional, and epistemic [56]. These aspects have gained prominence in research on digital health and mHealth intervention development [57,58] and in the context of mHealth interventions supporting parents during, for instance, pregnancy and postpartum, or in child development [36,59,60]. Most studies agree on the importance of the following components: (1) health promotion, (2) communication—both peer interaction and communication with health care professionals, (3) health tracking, (4) personalization, (5) notifications and reminders, (6) scientific foundation, and (7) patient education [37,41,61].

All these components also emerged as prevalent themes in this study and will be discussed in the following sections, supplemented by a few additional major themes that arose only in our study.

### Empowerment and Health Promotion

Most participants expected the app to inform parents about the comprehensive effects of CMT and guide them in using CMT elements to improve their own and their infant’s health actively. The combination of information and actionable instructions was seen as a way to increase motivation and foster a sense of self-efficacy, defined as perceived competence [62,63]. Beyond the anticipated increase in self-efficacy, across multiple themes, participants consistently perceived the mHealth intervention as a tool for empowerment in times of helplessness, with empowerment defined as gaining control over health-related decisions and taking actions [64]. This is particularly relevant in the NICU context, where parents often experience a profound loss of agency, and highlights the value of an intervention that helps parents feel in control of their child’s health and treatment. Indeed, parental empowerment is a key component of CMT [17,65,66]. It empowers parents, for instance, by promoting their sense of agency and self-confidence [17,67], reducing stress [17,20,68], and enhancing well-being, the parent-infant bond, and interaction quality [69,70]. This aligns with and extends the family-integrated care approach, which redefines parents as primary caregivers in the NICU rather than passive observers [16]. A previous randomized controlled trial suggests that this approach leads to improved outcomes for both preterm infants and their parents compared to standard NICU care [16] or even family-centered care [71,72].

However, while some participants of this study perceived the mHealth intervention as a solution to overcome hesitations to sing in a public space, previous research, which also observed this reluctance to sing in the NICU [70], emphasizes the essential role of guidance and support from music therapists. Similarly, other studies identified personal support complementary to the mHealth intervention as 1 out of 4 crucial factors for adherence to these interventions [73]. This suggests that a blended therapy approach might be needed at the beginning of the intervention, with the mHealth intervention complementing face-to-face interventions to receive the advantages from both [74].

### Design: Inclusivity, User-Centeredness, and Simplicity

Given the intervention’s intended use across diverse user groups, inclusive design is crucial to ensure accessibility and usability for individuals with different physical, cognitive, and social backgrounds. Moreover, participants highlighted the need for a user-friendly and intuitive design that simplifies interaction.

This is in line with previous research showing that successful technology adoption and uptake depend on perceived costs, such as cognitive costs and self-efficacy beliefs [75,76]. Complex mHealth apps that are complicated to navigate can increase cognitive costs, reduce self-efficacy beliefs, and ultimately reduce the users’ willingness to engage with them.

### Functions: Communication, Notifications, and Reminders

Participants clearly preferred an app that includes interactive elements enabling communication with health care professionals and peer-to-peer support. This interest in integrating social interaction and emotional support in mHealth tools aligns with previous studies, which found that both are crucial for engagement in all kinds of mHealth interventions [77,78]. Such features have been shown to foster feelings of support and connectedness, particularly when users perceive that someone has taken the time to send messages and engage with them [79]. Nevertheless, it indicates again that a blended therapy approach might be warranted to preserve the unique benefits of face-to-face communication [74].

Another required function was notifications and reminders. Both are generally seen as helpful for improving user engagement and adherence to mHealth interventions. However, it appears crucial that notifications are tailored to individual progress and specific user needs—for instance, in terms of their schedule and routines, behaviors and preferences—rather than providing generic or frequent alerts, which can become annoying or be ignored [73,80,81]. Furthermore, the reminders’ language has been shown to play a role [80].

Reminders are not the only form of notifications, which might be worth implementing in an mHealth intervention. Preliminary evidence suggests that theory-driven trigger messages based on behavior change theories can have beneficial effects on user engagement by providing reinforcement and targeting motivation, self-efficacy, knowledge, and self-care [81].

### Personalization

Personalization emerged as a cross-cutting theme across multiple topics, particularly regarding design and functionalities. For instance, participants would like the app to provide customized content, such as a tailored introduction to and progression through the use of music. This is consistent with other research on mHealth interventions, which shows that personalized designs and functions lead to greater satisfaction, long-term adherence, and effectiveness [73,77,82,83]. Gosetto et al [82] described 4 dimensions of personalization of mHealth: users, system functionalities, information, and app properties. All these aspects were also mentioned by participants, either explicitly or implicitly, in the context of personalization.

### Content: Scientific Foundation, Education, and Health Tracking

Regarding content, participants emphasized the importance of providing scientifically validated information to ensure the intervention’s credibility and trustworthiness and, consequently, a reduction of stress. This suggests that participants viewed the app not only as a tool for using music for health but also as a means of transmitting medical and health education. Furthermore, clear instructions and tutorials on how to use the app and interpret the information were seen as crucial for facilitating usability. This finding aligns with previous literature underlining the importance of mHealth interventions with high standards of content quality, ensuring that information is not only accurate but also understandable and actionable (eg, [84]). Additionally, increasing parents’ knowledge on essential aspects of preterm birth can improve their decision-making, which, in turn, affects maternal and infant health [40].

Thereby, to foster adherence to the intervention, it appears especially important to emphasize the positive effects of music therapy [19,52,85] and kangaroo care alone [14] on parents and preterm infants as well as the additional effects stemming from a combination of both [70,86].

Furthermore, incorporating a diary option was frequently mentioned as a valuable tool for parents. This might be explained by the positive impact of narrative diaries in the NICU, which has already been shown [87]. According to a systematic review, these diaries can “improve humanization, communication between NICU staff and parents, parents’ coping, and closeness to their newborn” [87]. A diary as part of an mHealth intervention might have similar promising effects. Another advantage is that they can easily be integrated into existing routines, which, as mentioned above, might enhance an mHealth intervention’s acceptance. Diaries are already implemented in many NICUs and could just be expanded by adding music and sounds next to pictures of the infants.

### Sustainability and Long-Term Use

The concept of sustainable, long-term use emerged as another critical topic. Previous research indicates that music therapy generally has potential for long-term use and impact on preterm infants and their families, even after NICU discharge [70,88,89]. For instance, combining music with reading has been shown to improve language development in preterm infants at 2-3 years of age [89], and music became an integral part of their families’ daily life even after supervised interventions ended [70].

Many participants indicated that the long-term use of mHealth interventions depends on the app’s usability across different stages of the child’s development. As mentioned earlier, personalization in the form of tailored, acutely relevant content, functions, and design might play a key role in long-term intervention adherence. Previous research supports the importance of personalized design, functions, and knowledge transfer and adds personal goals as crucial factors for long-term application of mHealth interventions [83].

Therefore, an mHealth intervention integrating music therapy elements, ongoing support, tailored information, personalization, and coaching is likely to enhance the intervention’s sustainability, long-term use, and overall effectiveness.

### Acceptance: Addressing Diverse User Needs, Updates, Data Security

Acceptance emerged as a recurring theme, with participants—in particular health care professionals—underscoring the importance of addressing the diverse needs of both parents and clinical staff, particularly given the time constraints faced by the latter. This demonstrates that a human-centered design is imperative, considering the perspectives and daily realities of both as well as the context of use from early on in the design process [45], thereby reinforcing the value of the ECOUTER approach’s participatory principles. In addition, participants expressed the need for regular updates to ensure that the app content remains relevant and engaging. This is also reflected in their desire for continuous improvement and responsiveness to users’ needs. Furthermore, data security was identified as a critical concern. Participants stressed that ensuring privacy and protection of personal health data would significantly influence their willingness to adopt and continue using the app. This aligns with existing literature on digital health adoption, which emphasizes privacy concerns as a considerable barrier to the widespread use of health technologies (eg, [79,90]). Interestingly, in other studies on digital interventions, this concern was not shared by patients or therapists [91], suggesting that perceived risks may vary depending on the type of data involved.

### Strengths and Limitations

Given the increasing focus on family-centered and family-integrating care in neonatology, involving family members of preterm infants in developing this mHealth intervention appears crucial for ensuring their needs are met. At the same time, experts from a range of health care professions could contribute valuable expertise and insights into the needs of NICU staff and how a CMT-based intervention may be integrated into the dense and demanding setting of NICUs. Thus, including both groups of participants and the diversity of their roles is a clear strength of this study. Moreover, the large number of contributions, their level of detail and their diversity—reflecting the broad spectrum of perspectives—along with continuous coediting and iterative restructuring led to a comprehensive framework of user needs and preferences for a usable and acceptable mHealth intervention. Saturation was assumed once no new input emerged, and participants confirmed that nothing more could be added. This demonstrates that following the ECOUTER approach was both meaningful and effective. Furthermore, as the approach is based on iterative feedback loops, it allowed us to conduct multiple rounds of participant engagement and to focus on purposeful sampling for the final round, specifically recruiting scientific advisory board members, experts, and parent representatives from premature infant organizations.

However, several limitations of this study should also be acknowledged, primarily related to the study sample. First, while the qualitative design and iterative ECOUTER-informed engagement enabled in-depth exploration and yielded convergent themes across multiple rounds of participant input, the overall sample size was small, which may limit the transferability of the findings. However, the ECOUTER approach facilitated sustained, reflective engagement and enhanced analytic depth, partially mitigating constraints typically associated with single focus group designs [48]. In addition, participants were purposefully selected to represent a range of relevant stakeholder perspectives (including parents, clinicians, music therapists, and psychologists), which strengthened the relevance of findings for intervention development but also resulted in a sample with a high overall educational level. As all participants had completed tertiary education, the perspectives of people with lower socioeconomic status and lower health literacy may be underrepresented, potentially limiting applicability to more socioeconomically diverse populations due to potential differences in information needs, comprehension, and access. Furthermore, participants were recruited from several countries; however, these countries are culturally comparable, and the study did not aim to examine or compare health care systems. While contextual differences may still have influenced individual perspectives, this relative cultural similarity supports thematic coherence. Nonetheless, these factors should be considered when interpreting the findings and applying them to the development of an mHealth intervention for parents.

Second, the questions posed during the focus groups were primarily formulated by FBH. Her professional background and experience as a music therapist in the NICU might have influenced them. However, the question guide was cocreated with other interdisciplinary members of the core research team, and all discussions were held and documented by several interdisciplinary researchers.

Third, this study adopted a real-world, pragmatic approach based on an online collaboration tool. Due to this and anonymization requirements, the contributions on the digital board could not always be attributed to individual participants. Meanwhile, the online format of focus groups may have limited spontaneous, dynamic discussions that are typically seen in in-person settings and are characteristic of group processes. Prior research suggests that virtual focus groups may hinder nuanced dialogues and the ability to pick up nonverbal communication cues (eg, [92,93]). However, these ambiguities were considered a trade-off in favor of the flexibility the online format offers in terms of time and location.

Finally, while stickers allowed participants to visually emphasize certain aspects of the mind map, this method may have created the impression of a consensus or dominant views. Items marked more frequently might be perceived as more important than they are, and it might have biased those prioritizing later. Despite this potential bias, the approach provided valuable insights into participants’ priorities and highlighted the path for future developmental work.

### Conclusion and Implications

To our knowledge, this is the first study that provides in-depth insights into the needs, expectations, and concerns of parents and health care professionals regarding a CMT-based mHealth intervention in neonatal care. The NICU—characterized by emotional intensity, parental vulnerability, and clinical pressure—requires particularly sensitive and well-integrated digital solutions. Findings from this study highlight the importance of a user-centered and participatory design approach, engaging diverse user groups, to respond to their individual needs and ensure emotional sensitivity as well as clinical acceptability in the NICU.

The results show that the proposed CMT-based mHealth intervention is seen as a meaningful complement to traditional CMT, which ensures wider access, promotes equity, and fosters parental engagement and empowerment in a situation often marked by helplessness. Key user needs toward the intervention include a simple and inclusive design, personalization, interaction possibilities with health care professionals and peers, and the integration into the daily realities of users. Addressing those needs is critical for the mHealth intervention’s acceptance and effectiveness.

Findings from this study provide a unique, valuable foundation for developing and codesigning the intervention. Subsequently, following a UCD approach, the intervention will be tested for usability, feasibility, and effectiveness, with each research step followed by system refinements before advancing to the next step [42].

## Appendix

Multimedia Appendix 1 Final version of the mind map structured in 5 themes and subthemes.

## Abbreviations

CMT: creative music therapy
COREQ: Consolidated Criteria for Reporting Qualitative Research
ECOUTER: Employing Conceptual Schema for Policy and Translation Engagement in Research
mHealth: mobile health
NICU: neonatal intensive care unit
R&D: research and development
UCD: user-centered design

## Data Availability

The raw data, including the different stages of map development, supporting the conclusions of this study, will be made available by the authors without undue reservation to any qualified researcher upon request.
