# Supplementary material for: Developing a Music-Based Mobile Health Intervention for Parents and Staff in Neonatal Care and Beyond: Online Focus Group Study
Source: JMIR Pediatr Parent. 2026 Jul 29;9:e85344. doi: 10.2196/85344 (PMC13469829; doi:10.2196/85344)
Supplement: Supplementary file 1 [file pediatrics_v9i1e85344_app1.docx]

**Multimedia Appendix 1.** Final version of the mind map structured in 5 themes and sub-themes.

Note: Red sticky notes indicate prioritized aspects; black sticky notes indicate potentially critical aspects or aspects requiring caution.
